# The Mitochondrial Genome of a Freshwater Pelagic Amphipod *Macrohectopus branickii* Is among the Longest in Metazoa

**DOI:** 10.3390/genes12122030

**Published:** 2021-12-20

**Authors:** Elena V. Romanova, Yurij S. Bukin, Kirill V. Mikhailov, Maria D. Logacheva, Vladimir V. Aleoshin, Dmitry Y. Sherbakov

**Affiliations:** 1 Laboratory of Molecular Systematics, Limnological Institute, 664033 Irkutsk, Russia; bukinyura@mail.ru (Y.S.B.); sherb@lin.irk.ru (D.Y.S.); 2 Belozersky Institute for Physicochemical Biology, Lomonosov Moscow State University, 119991 Moscow, Russia; kv.mikhailov@belozersky.msu.ru (K.V.M.); maria.log@gmail.com (M.D.L.); aleshin@genebee.msu.su (V.V.A.); 3 Institute for Information Transmission Problems of the Russian Academy of Sciences, 127994 Moscow, Russia; 4 Center of Life Sciences, Skolkovo Institute of Science and Technology, 121205 Moscow, Russia; 5 Department of Information Biology, Novosibirsk State University, 630090 Novosibirsk, Russia

**Keywords:** long mitochondrial genomes, non-coding regions, direct and inverted repeats, gene duplications, amphipods, Lake Baikal

## Abstract

There are more than 350 species of amphipods (Crustacea) in Lake Baikal, which have emerged predominantly through the course of endemic radiation. This group represents a remarkable model for studying various aspects of evolution, one of which is the evolution of mitochondrial (mt) genome architectures. We sequenced and assembled the mt genome of a pelagic Baikalian amphipod species *Macrohectopus branickii.* The mt genome is revealed to have an extraordinary length (42,256 bp), deviating significantly from the genomes of other amphipod species and the majority of animals. The mt genome of *M. branickii* has a unique gene order within amphipods, duplications of the four tRNA genes and *Cox2*, and a long non-coding region, that makes up about two thirds of the genome’s size. The extension of the mt genome was most likely caused by multiple duplications and inversions of regions harboring ribosomal RNA genes. In this study, we analyzed the patterns of mt genome length changes in amphipods and other animal phyla. Through a statistical analysis, we demonstrated that the variability in the mt genome length may be a characteristic of certain phyla and is primarily conferred by expansions of non-coding regions.

## 1. Introduction

Mitochondrial (mt) genome sequencing is a powerful tool used in many areas of modern biology, such as phylogenetics and phylogenomics; population genetics and molecular evolution; and studies of biodiversity, conservation, aging, and genetic diseases. At the same time, a mitochondrial genome is itself used for the investigation of fundamental molecular mechanisms governing its functionality and evolution [1,2].

Previous studies have demonstrated that most animal phyla have a relatively uniform mt genome length and gene content, thus establishing the concept of a “typical mt genome” in animals [3]. Indeed, the majority of animal mt genomes are single circular molecules of about 16 Kbp, with 13 protein-coding genes (PCG), that encode components of the electron transport chain and ATP synthesis, 22 tRNA genes, 2 ribosomal RNA genes, that provide a basis for the in-house protein synthesis machinery, and a control region, that maintain regulatory elements for replication and transcription [4]. However, deeper exploration of animal diversity, facilitated by advances in sequencing technologies, have shown that the mt genomes of many species deviate in terms of structure, length, gene content, and gene order from the archetypical animal mt genome [3,5,6].

For instance, some lineages of Cnidaria and Porifera possess linear mt genomes, that may occur as a single chromosome or be partitioned into multiple chromosomes [7,8,9]. Representatives of nematodes, insects, and rotifers have mt genomes encoded on several circular chromosomes [10,11,12].

The mt genomes of many species possess additional mt genes and/or non-coding regions acquired as a consequence of duplications [13,14]. For instance, duplications of one or several PCGs and ribosomal genes were found in mollusks [15,16,17] and nematodes [18] etc., while tRNA gene copies are frequently found in lengthy mt genomes of bivalves [19,20], freshwater sponges [21], and typical mt genomes of most Baikalian amphipod species [22]. Copies of control region are found in salamanders of the gen. *Aneides* [23], as well as in some bird taxa [24,25,26].

Although it has been suggested that one of the duplicated segments rapidly accumulates mutations and eliminates from the genome [6,13], there are many examples of taxa where duplicated segments exist for a long time [17,21,23,27].

The mt genomes of a few animal taxa have been shown to have PCGs beyond the “typical” mt gene set. Among the most common of these is *Atp9*, a gene for subunit 9 of mitochondrial ATP synthase, which is found in the mt DNA of sponges [3,28,29,30]. *PolB*, coding a putative DNA-directed DNA polymerase type B of the fungal origin, was found in the mt genomes of Medusozoan Cnidarians [31] and some Placozoan taxa and assumed to be acquired through horizontal gene transfer [32]. A homolog of the bacterial *MutS*, a component of the bacterial DNA mismatch repair pathway [33], was found to be transferred to the ancestral mt genome of Octocorallia [34]. The differential expression of alternative transcripts of this gene was shown in an octocoral *Sinularia* cf. *cruciate* [35], suggesting a functional requirement for this gene in the studied species.

The mt genomes of many organisms contain open reading frames (ORFs) with unknown origins and functions (referred to as mtORFans) but experimentally proven expression [36]. Such sequences have been found in corals from the family Pocilloporidae (*tmp362*) [37], sea anemones *Anemonia viridis* and *Anemonia majano* (*orfA*) [38], insects of gen. *Campsomeris* (Scoliidae) (*qnu*) [39], Bivalvia mollusks (F- and M-orf) [40], etc., and assumed to be regular PCGs [37,39] or pseudogenes affecting the expression of nuclear genes [41,42,43].

Along with gene acquisition, many cases of gene loss have been detected in animal mt genomes. For example, the absence of *Atp8* has been shown in many Platyhelminthes [44,45] and bivalves [20,46], as well as for nematodes [47], Hexactinellida sponges [48], and many other taxa. Representatives of the subclass Octocorallia [35,49] and several lineages of the phylum Porifera [30,48] have lost the majority of their tRNA genes. Probably the most drastic cases of mt genome reduction have been noted in small marine invertebrates from the phyla Chaetognatha (arrow worms) [50,51] and Ctenophora (comb jellies) [52,53], where the shortest mt genome sequences currently known are present in Ctenophora *Mnemiopsis leidyi* (10,326 bp) [54] and Chaetognatha *Sagitta enflata* (12,631 bp) [51]. The mt genomes of both taxa lack *Atp6* and *Atp8* and all tRNA genes, and only some Ctenophora species retain the *trnM*(cau) [51,52,53,54]. In some cases, however, previously missing PCGs and tRNA genes can be found in mt genomes after the application of more sensitive annotation techniques or through additional experimental studies [22,55,56,57,58,59].

There are many animal species and lineages where mt genomes greatly exceed the average length of 16–20 Kbp. The largest singular mt genomes are shown in Bivalvia mollusks from the Arcidae family [15,19,20,60] (more than 40 Kbp in length), Placozoan species (from 23,462 to 43,079 bp) [61,62], and freshwater sponges of the order Spongillida (from 23,929 to 28,958 bp) [21,30,63]. The adaptive significance of variations in the lengths of mt genome in different organisms and taxa is a subject of ongoing research.

It is anticipated that the evolution of mt genome characteristics, such as the overall genome size, complies with the fundamental rule of population genetics [64] and depends on the mutation accumulation rate and the power of genetic drift (inversely proportional to the effective population size) [65,66,67]. The main mechanisms of sequence extension are slipped strand mispairing and errors in termination during mt genome replication [68,69]; however, more specific mechanisms, such as transposition, retrotransposition via an RNA intermediate, and recombination, were suggested to explain the proliferation of repeat elements in mt genomes and contribute to size expansion [68,69,70,71,72]. Purifying selection removes redundant genes and non-coding fragments, favoring compact mt genomes in most lineages [73,74], as shorter genomes are thought to have more effective transcription and replication [73], while excessive DNA is a target for deleterious mutations [67]. On the other hand, many researchers assume there to be no association between the excessive mt genome length conferred by selfish elements and negative organism fitness, which is corroborated by studies on *Drosophila melanogaster* laboratory lineages [75], freshwater sponges [21], and salamanders of *Aneides* spp. [23]. Recent findings have shown that such selfish elements may increase the replicative potential of certain mtDNA sequences and lead to the positive selection of such variants [66,67,76].

There is a growing body of evidence showing that short and long non-coding RNAs (ncRNA) transcribed inside known ORF (PCGs), ribosomal genes, intergenic regions, and pseudogenic sequences of nuclear and mt genomes participate in the regulation of different processes, such as protein translation, RNA methylation and splicing, mRNA degradation and silencing, etc. [77,78,79]. Translated short ORFs (30–60 bp) encode biologically active peptides that may also have regulatory functions in cells, and some of those peptides (humanin, SHLP 1-SHLP 6, MOTS-c) originate from the mt genome [78]. Data on the functionality of mt ncRNA and peptides obtained in human and murine cells models suggest the existence of similar regulatory units in the mt genomes of non-model species with non-canonical mt genes or inside their non-coding regions [5,80].

The sequencing and analysis of mt genomes with unusual lengths and peculiar architectures will help in studying the evolution of these features and provide direction for further researching the processes of maintenance and regulation in mitochondria.

Amphipods of the ancient Lake Baikal are a useful model for studying different aspects of mt genome evolution, as previous studies have shown many peculiarities in their mt DNA [22,81]. We found an unusual variability in mt genome lengths (from 14,370 to 18,114 bp) and gene orders within the currently sequenced mt genomes of ten representatives. Further analyses have revealed an unusually high number of tRNA genes that have undergone duplication and remolding (changes in tRNA gene identity through singular or multiple mutations in anticodon sequence) in the mt genomes of Baikalian species in comparison to those of amphipods from other habitats [22]. Out of more than 350 Baikalian amphipod species, *M. branickii* (Dyb.) is the only pelagic amphipod dweller [82,83]. This species inhabits the whole lake and is usually encountered at depths of more than 100–300 m. [84,85]; however, specimens are sometimes found at shallower depths and even at the water’s edge [86,87], as the species performs diel vertical migrations from deep to shallow water layers [82,88]. *M. branickii* is an important component of the lake ecosystem; it is the main zooplankton predator, as well as an essential feeding component of pelagic fishes (gen. *Comephorus*, gen. *Cottocomephorus*, *Coregonus autumnalis migratorius*) [88].

In this study, we describe the mt genome of *M. branickii*, detail its extraordinary length, and discuss the mechanisms of this extension. In the context of this finding, we analyze the mt genome length distributions in different phyla of invertebrate animals to reveal how frequently variations in mt genome length occur and try to find common and distinctive features in their architectures.

## 2. Materials and Methods

### 2.1. Sampling, DNA Sequencing and Assembly

*M. branickii* samples were collected in 2015 at the south basin of Lake Baikal near the estuary of Harauz river (52°0′24″ N, 105°59′04″ E) at a depth of 0–70 m. Amphipods were attracted during the nighttime by the artificial light of the research vessel “G. Titov” and collected using a Juday plankton net. Total DNA was extracted from separate individuals using the modified CTAB method [89].

The genomic sequencing of a single individual of *M. branickii* was performed at the Faculty of Bioengineering and Bioinformatics of Lomonosov Moscow State University with an Illumina HiSeq 4000 system. A total of 41 million 150 bp paired-end reads were generated. The reads were cleaned with Trimmomatic [90] to remove sequencing adapters and assembled with SPAdes [91] using k-mer sizes of 21, 33, 55, 77, and 99. Mitochondrial sequences were detected in the assembly by BLAST [92] searches with the mtDNA-encoded protein sequences of amphipods. Fragmented mt contigs were extended by iteratively aligning read pairs to the ends of contigs. We used BLAST searches with the ends of contigs to find corresponding reads in the sequencing library and then aligned read pairs to the contig, thus extending its sequence. The sequences were extended until sufficient overlaps with other mt contigs were available. The contigs were then merged using overlapping sequences while manually resolving cases of inverted repeat structures.

### 2.2. Mt Genome Sequence Verification and Annotation

To validate the mt genome assembly obtained from total genomic reads, we additionally performed an assembly with transcriptomic data of *M. branickii* acquired from the Sequence Read Archive SRR3467077 [93]. Assembly was performed with SPAdes in single-cell mode (—sc) using k-mer sizes of 55 and 77. Mt contigs from both assemblies were aligned and inspected manually using BioEdit [94].

The merges and long inverted repeat regions in the assemblies were additionally verified by PCR and Sanger sequencing and by mapping reads to the complete mitochondrial sequence. The areas containing prominent repeats were amplified in two fragments of about 5 Kbp and 2 Kbp, and then sequenced using the primer walking method. Read mappings were performed using Bowtie2 [95] with the genomic and transcriptomic sequencing libraries. The mappings were inspected in Tablet [96] and visualized as circular diagrams using Circos [97]. The coverage statistics for genes were obtained from the read mappings using the BEDTools [98] genomecov utility. Duplicated reads were excluded from the mapping using Picard Tools (http://broadinstitute.github.io/picard) (accessed on 20 January 2021). Histograms of coverage were built from the sequence mappings with coverage values calculated using the rolling average in a 50 bp sequence window.

The mt genome sequence of *M. branickii* was annotated using the MITOS pipeline [99]. The prediction of the tRNA genes was performed with MiTFi [13] using both the default metazoan covariance models and the amphipod-specific models developed in our previous study [22]. The secondary structure visualization of tRNAs was carried out using the forna package [100]. The *M. branickii* mt genome map was also visualized using the OGDRAW program [101]. PCGs and ribosomal gene boundaries were manually corrected using sequence alignments with genes from the previously published amphipod mt genomes.

### 2.3. Structural Analyses of M. branickii Mt Genome and Phylogenetic Inference of Amphipods

Basic statistics for the nucleotide content of the newly sequenced mt genome were calculated using BioEdit [94].

Direct and inverted repeats in the mt genome sequence of *M. branickii* and four other Baikalian amphipods with length >17 Kbp (*Acanthogammarus victorii*, *Brachyuropus grewingkii*, *Garjajewia cabanisii*, *Gmelinoides fasciatus*) were found using NUCmer (-l 10 —maxmatch —nosimplify) and visualized using the Mummerplot of the MUMmer3.23 package [102]. To define the content of non-coding sequences of mt genomes from listed Baikalian species, we annotated the ORFs in these regions using the online version of the ORF finder integrated with the NCBI database (https://www.ncbi.nlm.nih.gov/orffinder/, accessed on 7 March 2021). ORFs with a minimal length of 30 nt. were found using the invertebrate mt genetic code and translated amino acid sequences were used for carrying out blastp searches in the online version of BLAST with using default settings. BlastN search with the non-coding regions was conducted with standalone BLAST (v.2.6.0.) using previously published amphipod mt genomes as queries (*Eulimnogammarus vittatus* KM287572, *Pallaseopsis kesslerii* KX341968, *Gammarus duebeni* JN704067, *Metacrangonyx repens* HE860495, *Caprella mutica* GU130250, *Parhyale hawaiiensis* MH542432). The analysis was conducted using the following settings: -word_size 9 -gapopen 2 -gapextend 1 -reward 1 -penalty -1 -evalue 0.001.

To infer the taxonomic position of *M. branickii* within other amphipods, we built a Maximum likelihood phylogenetic tree using IQ-TREE v.1.6.9. [103] based on the concatenated alignments of the amino acid sequences of 13 mt PCGs, including the newly sequenced species, ten other Baikalian species, and some non-Baikalian amphipods. Individual mt PCG sets and deduced amino acid sequences were aligned using Mafft [104] implemented in the local version of the TranslatorX program [105]. The substitution model mtMet+F+R10 was selected for the amino acid dataset using ModelFinder [106] implemented in IQ-TREE [103]. The SH-aLRT test and ultrafast bootstrap with 3000 replicates were used to assess node support values [107,108]. The resultant tree was rooted with the outgroup species and visualized in FigTree v.1.4.3 [109].

### 2.4. Statistical Analysis of Mt Genome Sequences from RefSeq

A dataset of animal mt genomes was acquired from the RefSeq database (entries released before 1 January 2020) and processed to extract the mt genome lengths. The dataset included data from species with mt genomes organized as a singular “chromosome”. The lengths of the coding and non-coding parts of every mt genome were counted based on annotation data using custom R scripts. Animal mt genomes were separated into groups according to animal phyla from the Taxonomy Browser of the NCBI database. Phyla maintaining three or more species (Annelida, Arthropoda, Brachiopoda, Bryozoa, Chaetognatha, Chordata, Cnidaria, Ctenophora, Echinodermata, Hemichordata, Kinorhyncha, Mollusca, Nematoda, Nemertea, Onychophora, Placozoa, Platyhelminthes, Porifera, Sipuncula, Tardigrada, Xenacoelomorpha) were selected for further analysis.

Distributions of the mt genome length characteristics (length of the entire mt genome, length of the coding part, length of the non-coding part, and the ratio of lengths of non-coding part to coding part) of every animal phylum were visualized as boxplots. The outliers were defined as the values of the elements that were less from the first quantile of the distribution (downward outlier) and more than the third quantile of the distribution (upward outlier) according to a three-fold interquartile range (IQR). We used a 3-fold IQR to select a relatively small number of mt genomes with very diverse lengths for further analyses.

To test if the number of sequences in a phylum (sample size) significantly affects the mt genome length variability, we used regression analysis. The measures of the mt genome length variability in every phylum were the aforementioned characteristics (length of the entire mt genome, length of the coding part, length of the non-coding part, and the ratio of lengths of the non-coding part to lengths of the coding part) and the proportion of outliers. Additionally, a regression analysis was used to assess the contribution of the coding and non-coding regions to the length of the mt genomes by calculating the dependencies of the coding region lengths, non-coding region lengths, and their ratio on the length of the entire mt genome. We carried out all types of regression analysis with the next regression models: linear regression, second-degree polynomial, third-degree polynomial, exponential dependence, power dependence, and logarithmic dependence. The best regression model was chosen according to the Bayesian information criterion (BIC) (minimal BIC value for the best model) [110]. For the best regression model chosen, we calculated the R^2^ covariance coefficient and estimated its reliability using the F-test (F). We assumed that the regression model was trustworthy with a *p*-value threshold of 0.05. The regression analysis and visualization were conducted with the standard function set of the R programming language according to the guidelines detailed in Reference [110].

The nonparametric version of ANOVA was used to examine the dependence of the genome length characteristics. The calculation of the ANOVA *p*-value was carried out using the permutation test [111] in the «lmPerm» package for the R programming language. ANOVA was used to test the dependencies of the entire mt genome lengths of all available animal species (9127 species) for three subsets of the data: (i) a set of mt genome lengths within general distributions (excluding values from outliers); (ii) a set of mt genome lengths from downward outliers; (iii) a set of mt genome lengths from upward outliers. The analysis shows if there is a significant difference between the lengths of the mt genomes belonging to the outlier categories in every phylum and the lengths of the rest of the mt genomes of this phylum.

### 2.5. Phylogenetic Analysis and Analysis of Repeats in Selected Mt Genome Sequences from RefSeq

Species whose non-coding mt genome region sizes were identified as outliers were selected for phylogenetic analysis. The datasets included species of interest with long mt genomes and all other species from the same taxa available in the RefSeq mt genomes. The set of taxa for the analysis in every case was chosen for balanced taxonomic sampling. We did not analyze the mt genome sequences of the phylum Chordata in this study because of their high number and the prevalence of small mt genomes without extensive non-coding regions in this group.

Substitution saturation tests were performed for the 1st + 2nd codon positions of each mt PCG in every sequence set using DAMBE v.7.2.43 [112]. Amino acid sequences from genes without saturation were concatenated and used for phylogenetic inference. The taxa used in the analysis, selected gene/protein sets, and the substitution model used in every set are summarized in Table S1. Phylogenetic trees were built and visualized using the aforementioned software.

The repeats in mt DNA sequences from the outlier set were examined and visualized using the pairwise alignment utility of the online version of BLAST.

## 3. Results

### 3.1. M. branickii Mt Genome Assemblies and Features

**Figure 1:**
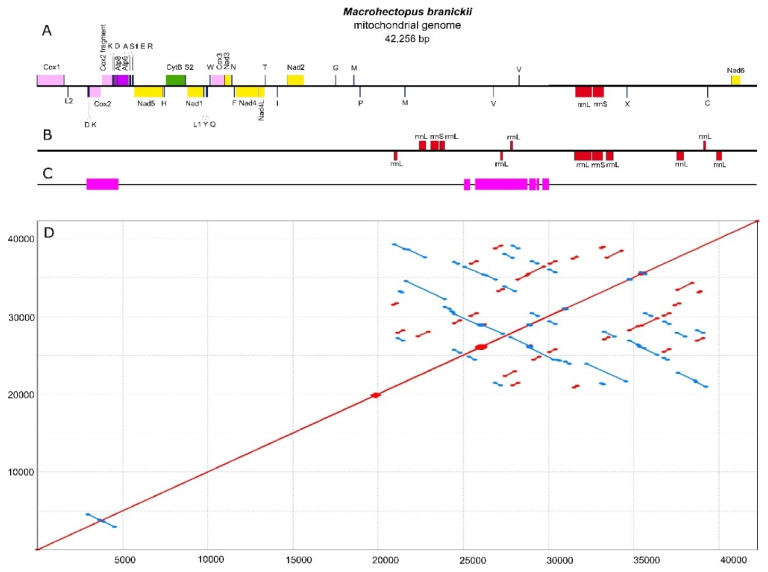
(**A**) Linear map of the mt genome of *M. branickii*; genes encoded by the positive strand are located above the line and genes encoded by the negative strand are located below the. tRNA genes are labeled by their single-letter amino acid code. X is a tRNA pseudogene with a CCCC sequence in its anticodon. (**B**) Ribosomal RNA gene fragments (red) found using BLAST search in a long non-coding region of the *M. branickii* mt genome. (**C**) Regions of the mt genome sequenced with the Sanger method (magenta). (**D**) A dotplot of repeat sequences identified by nucmer; red and blue lines indicate direct and inverted repeats, respectively.

**Figure 2:**
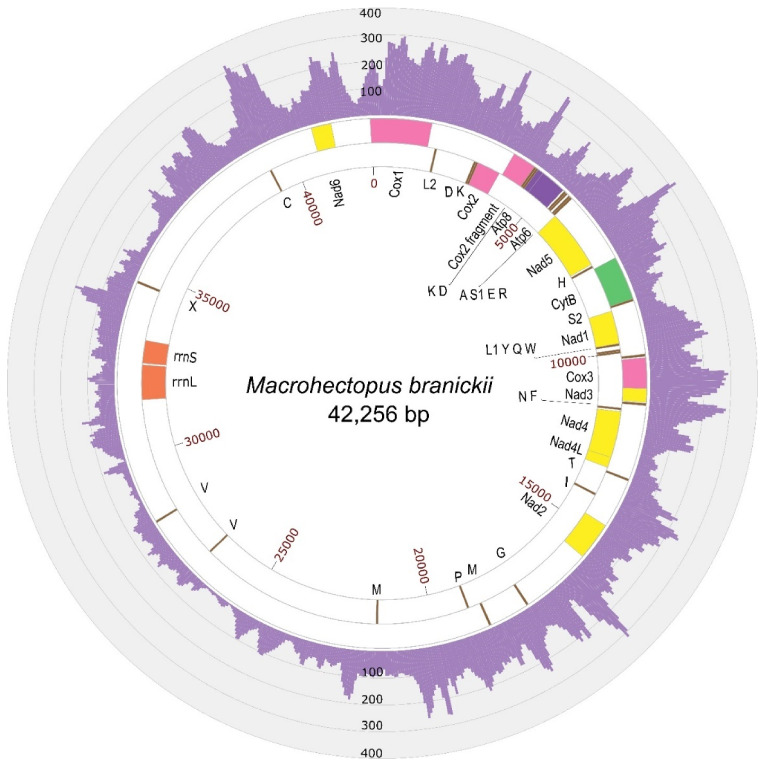
A circular map of the mt genome of *M. branickii* with histograms of genomic reads’ depth. Genes encoded by the positive chain strand are shown in the outside ring, while genes encoded by the negative chain are shown in the inner ring. tRNA genes are labeled by their single-letter amino acid code. X is a tRNA pseudogene with a CCCC sequence in its anticodon.

**Figure 3:**
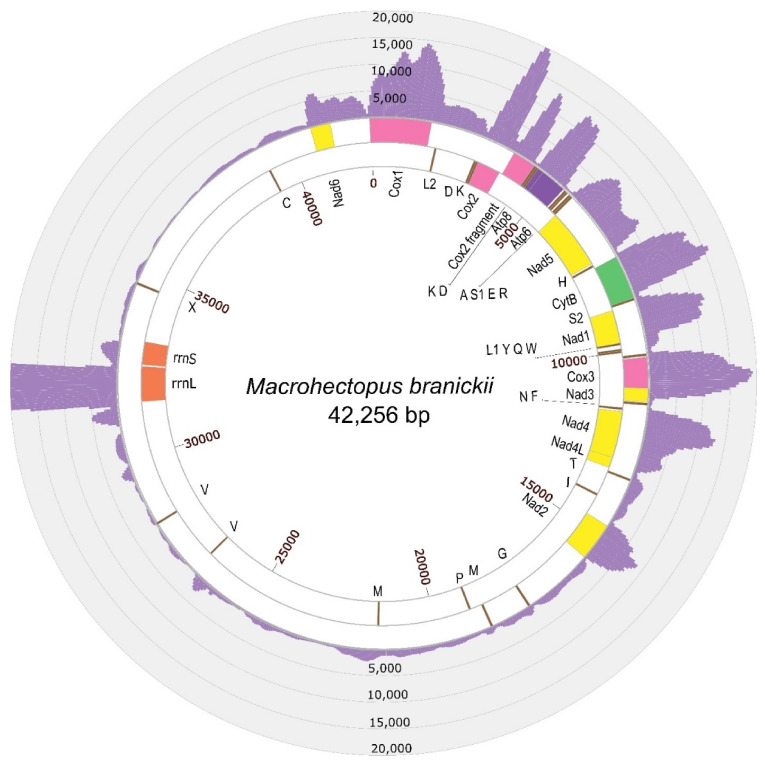
A circular map of the mt genome of *M. branickii* with histograms of transcriptomic’s reads depth. The maximum coverage value was set to 20,000 for the illustration; the coverage of the *rrnL* region reached 66,656 reads per nucleotide (Table S2). Genes encoded by the positive strand are shown in the outside ring, while genes encoded by the negative chain are shown in the inner ring. tRNA genes are labeled by their single-letter amino acid code. X is a tRNA pseudogene with a CCCC sequence in its anticodon.

Similarity searches with BLAST identified six mitochondrial contigs in the SPAdes assembly with the genomic sequencing data and another six contigs in the assembly with the transcriptomic data, ranging in size from 242 bp to 22 Kbp. Alignments and repeat resolution for mitochondrial contigs from each assembly resulted in a 42 Kbp circular sequence. The assembled sequence had two inverted repeat regions of 600 bp and 1.5 Kbp. These repeat regions were additionally verified by Sanger sequencing, generating six contigs with a total length of 6350 bp (Figure 1, File S1). For the amplification and Sanger sequencing, we used a DNA sample of the same amphipod individual that was previously used for the total genomic Illumina sequencing. The new sequencing data led to slight correction of the assembly, which turned into a final version of the *M. branickii* mt genome, spanning a total of 42,256 bp (GenBank accession MT047459). The average read depth of the genomic reads for the genome assembly was estimated to be around 120 reads per nucleotide (Figure 2, Table S2). The transcriptome read coverage was far less equal and was minimal in the non-coding part of the genome (Figure 3, Table S2).

The AT content of the total mt genome sequence is 59.20%. The AT-skew and GC-skew counted for the positive strand (coding the biggest portion of genes) of the entire mt genome of *M. branickii* were −0.0034 and −0.047, respectively, indicating a slight prevalence of pyrimidine over purine bases. The mt genome of *M. branickii* encodes 13 PCG, 2 ribosomal RNA genes, and 26 tRNA genes. All PCGs and the majority of the tRNA genes are grouped in a cluster that encompasses about 40% of the total mt genome length, while the ribosomal RNA gene cluster is separated from the PCGs by long non-coding regions spanning 16 Kbp and 7.5 Kbp (Figure 2 and Figure 3). The genes are distributed between two strands of the mtDNA: the rRNA genes and five PCGs are encoded on one (negative) strand, while the other eight PCGs are encoded by the opposite (positive) strand.

A duplicated fragment (559 bp) of the *Cox2* is located near the original *Cox2* (664 bp) in a reverse orientation, constituting a prominent inverted repeat unique to the coding cluster of mtDNA. The 559 bp copy is identical to the original gene but lacks the first 105 bp and, thus, is annotated as “*Cox2* fragment”. Both copies have adjacent *trnK*(uuu) and *trnD*(guc) near their 3′ ends, which implies that the *Cox2-trnK*(uuu)*-trnD*(guc) region is duplicated as a single unit.

The mt genome annotation in MITOS and a further BLAST search (Table S3) revealed partial copies of ribosomal RNA genes in the large non-coding segments between *Nad2* and *Nad6* (Figure 3). We defined the location of the true functional *rrnL* and *rrnS* based on the gene sequence integrity and the coverage values by transcriptomic reads (Figure 3, Table S2).

In the mt genome of *M. branickii,* we found additional tRNA gene copies along with the standard tRNA gene set. The Metazoan covariance models and amphipod-specific models predicted 26 and 28 tRNA genes, respectively (Table S4). One of the additional findings with the amphipod-specific models was *trnL2*(uaa) located in the region from 14,623 to 14,679 bp, which was ruled to be false positive due to its marginal bitscore (21.49) and e-value (6.79 × 10^−4^). The second finding was a tRNA gene located between 34,609 and 34,669 bp. This tRNA gene was identified using a model for methionine tRNA with an e-value of 5.89 × 10^−7^, but as its anticodon loop contained eight nucleotides with the CCCC sequence in the anticodon, we ruled this finding as a *trnM*(cau)-derived pseudogene and annotated it as *trnX*(cccc). The *M. branickii* mt genome has two identical copies of *trnV*(uac), two copies of *trnM*(cau) with an 88.5% identity, two copies of *trnK*(uuu) with 96.6% identity, and two copies of *trnD*(guc) with 90.0% identity. Each duplicated copy is located on the opposite strand. Their secondary structures were not impaired (Figure S1) and the transcriptome read coverage was comparable with the values of other singular tRNA genes (Table S2).

### 3.2. Non-Coding Regions of the Large Mt Genomes of Baikalian Amphipods

We annotated 64.8% of the 42,256 bp mt genome of *M. branickii* as non-coding regions. To analyze the content of non-coding regions in the mt genome of *M. branickii* and the mt genomes of other Baikalian amphipods with a total mt genome length exceeding 17 Kbp (*A. victorii*, *B. grewingkii*, *G. cabanisii*, *G. fasciatus*), we assessed the pattern of repetitive elements in these sequences and searched for additional ORFs and gene vestiges.

In the mt genome sequence of *M. branickii,* we found direct and inverted repeats ranging from 39 to 1632 bp which cover about 20 Kbp of the whole sequence. All repeat pairs were located in the large non-coding sequence between *Nad2* and *Nad6*, except for the *Cox2* duplication (Figure 1). Repeat searches in other Baikalian amphipods did not show such massive repeat expansions, even considering the differences in lengths of the non-coding parts (Figure S2).

The amino acid sequences translated from 633 ORFs predicted in the large non-coding sequences of the mt genome of *M. branickii* between *Cox1* and *Cox2*, *Nad2* and *Nad6*, and *Nad6* and *Cox1* did not produce any hits in Blastp searches against the nr/UniProtKB/SwissProt/refseq_protein databases. A similar BLAST search for translated ORFs from the non-coding parts of other mt genomes under consideration did not reveal homology with any protein either. Data on the ORF findings in the mt genomes of Baikalian amphipods are summarized in Table S5.

BLASTn searches revealed numerous copies of ribosomal RNA genes in the non-coding regions of the *M. branickii* mt genome (Figure 1) and three *Atp8* gene fragments of 81–83 bp predicted with a marginal e-value of 8 × 10^−4^ (Table S3). Short fragments of the *Cox2* (132 bp), *Atp8* (58 bp), and *Nad2* (64 bp) were detected with marginal e-values (from 1 × 10^−3^ to 6.5 × 10^−4^) in the control region of *B. grewingkii* and may constitute either degenerated gene copies or false-positive predictions. A truncated copy of the *CytB* of 405 bp was found in a non-coding region of *G. cabanisii* near the full-length *CytB*, indicating an event of duplication and subsequent degeneration. Additionally, small portions of the *rrnL* (100 bp), *Nad4L* (70 bp), and *Nad1* (115 bp) were detected in the non-coding regions between *rrnS* and *Nad2*. Truncated copies of the *Atp6* (135 bp) and *Nad4L* (197 bp) are found in a control region of *G. fasciatus*. No additional gene fragments were found in the non-coding regions of the *A. victorii* mt genome (Table S3).

### 3.3. Mt Gene Order of M. branickii and Its Phylogenetic Position within Baikalian Amphipods

**Figure 4:**
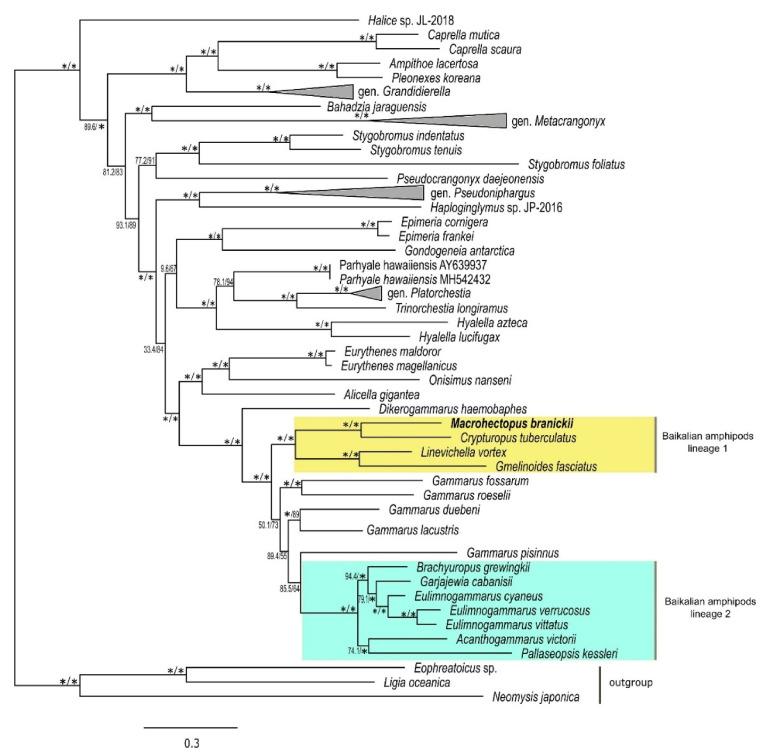
Maximum likelihood tree of amphipod species based on the amino acid alignments of 13 mt PCG sequences. Numbers on the branches indicate the percentage of ultrafast bootstrap and SH-aLRT, where values ≥ 95 are marked as asterisks. Yellow and blue rectangles show Baikalian amphipod species of the first and second lineages, respectively.

A Maximum likelihood phylogenetic tree based on the concatenated alignments of amino acid sequences of mt PCGs placed *M. branickii* inside a well-supported clade comprising one of the two lineages of Baikalian amphipods (Figure 4). This lineage combines small species that mainly inhabit shallow water and have a tolerance to high temperatures [83,113,114]. The placement of *M. branickii* in this amphipod lineage corroborates the results of previous studies based on nuclear molecular markers and the analysis of morphological features [93,115].

The mt genome of *M. branickii* has an unusual gene order that differs from patterns seen in the majority of sequenced Baikalian amphipods or the closest non-Baikalian species of gen. *Gammarus*. The gene order of the *M. branickii* mt genome mostly resembles the one of *Crypturopus tuberculatus*, the nearest species it clusters with. It is worth noting that the mt genomes of both *C. tuberculatus* and *M. branickii* have pseudo tRNA genes that originate from *trnM*(cau) duplications. The presence of additional tRNA genes found in the *M. branickii* mt genome may be regarded as a common feature observed in the majority of currently sequenced genomes of Baikalian amphipods [22].

### 3.4. Statistical Analysis Reveals Genome Length Modes in Invertebrate Phyla

Statistical analysis was performed for 9127 sequences of complete mt genomes of animals from the RefSeq database submitted before 1 January 2020 (accessed on 20 October 2020) (Table S6).

**Figure 5:**
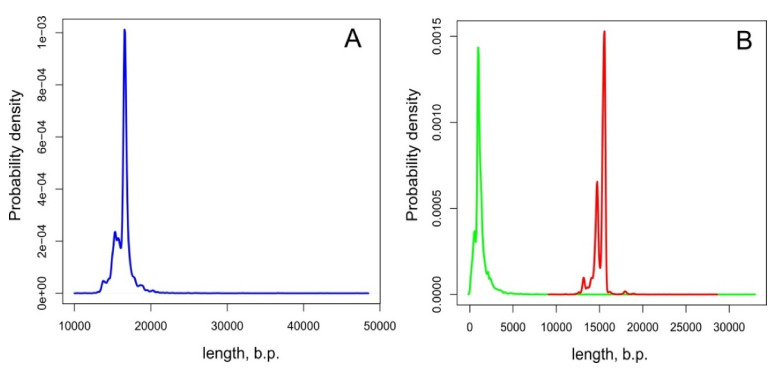
Distributions of animal mt genome lengths from the RefSeq database. (**A**) Distribution of total mt genome lengths. (**B**) Distribution of mt genome coding region lengths (red plot) and distribution of mt genome non-coding region lengths (green plot).

The distributions of total mt genome lengths and their coding and non-coding lengths have distinct peaks, indicating that the majority of the total mt genome lengths and their constituents vary within narrow ranges (Figure 5). At the same time, the long distribution tails indicate that a small number of values deviate significantly from the general averages in every group.

**Figure 6:**
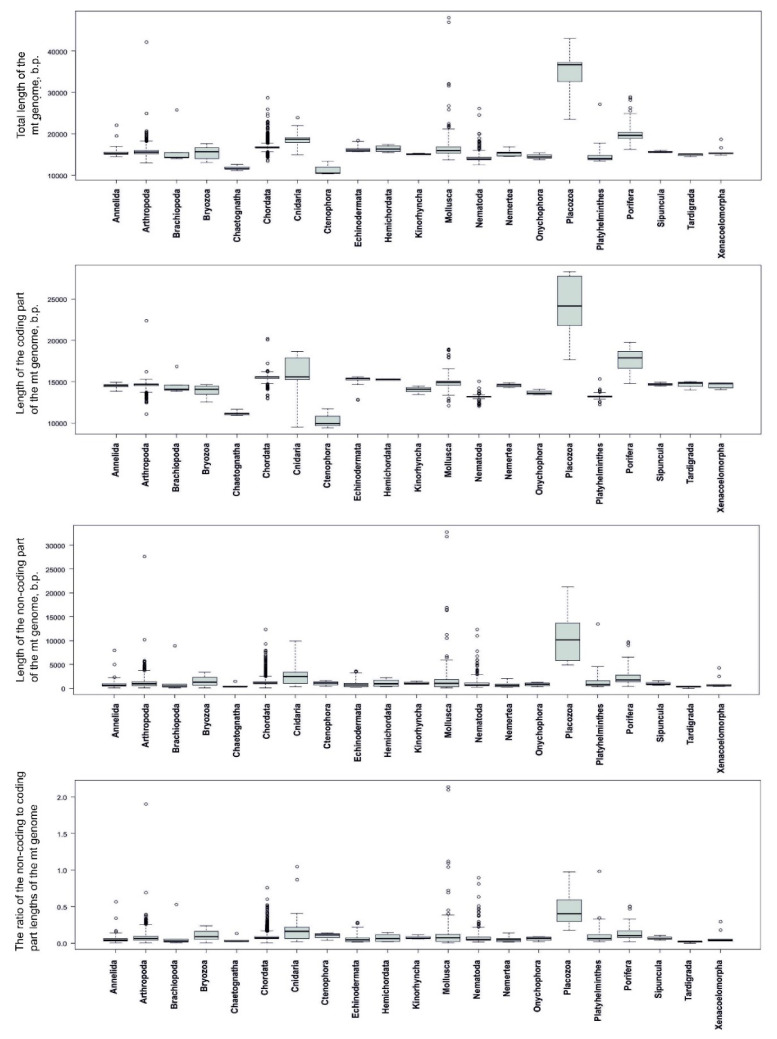
Distributions of the mt genome lengths in animal phyla.

Further statistical analyses of the mt genome length characteristics (lengths of the entire mt genome, coding lengths, non-coding lengths, and the ratio of lengths of non-coding to coding portions of the genomes) were performed individually for each animal phyla. Distributions of the sequence length characteristics for every phylum are shown as boxplots in Figure 6. Most plots of the different length characteristics of phyla are skewed, indicating that the distributions deviate from normal. The IQR values for the distributions of different phyla vary significantly within each other in groups with different length characteristics. Distributions of genome lengths in Placozoa have the biggest IQRs within the phyla in all the length characteristics groups. It is notable that the distributions of the non-coding lengths and the distributions of the ratio of lengths of non-coding to coding portions possess only upward outliers, whereas the distributions of the coding lengths in some phyla (Arthropoda, Chordata, Mollusca, Nematoda, and Platyhelminthes) have both upward and downward outliers. The mean values, standard deviations, and number of outliers were estimated for every phylum (Table S7) and used in a regression analysis. The RefSeq numbers of mt genomes that fall into the outliers category are shown in Table S8.

A regression analysis showed no dependence of the mt genome length characteristics on the number of sequences in a phylum (*p*-value from 0.28 to 0.70) (Table S9), as well as no dependence of the proportion of outliers in distributions on the number of sequences in a phylum (*p*-value from 0.39 to 0.87) (Table S10).

**Figure 7:**
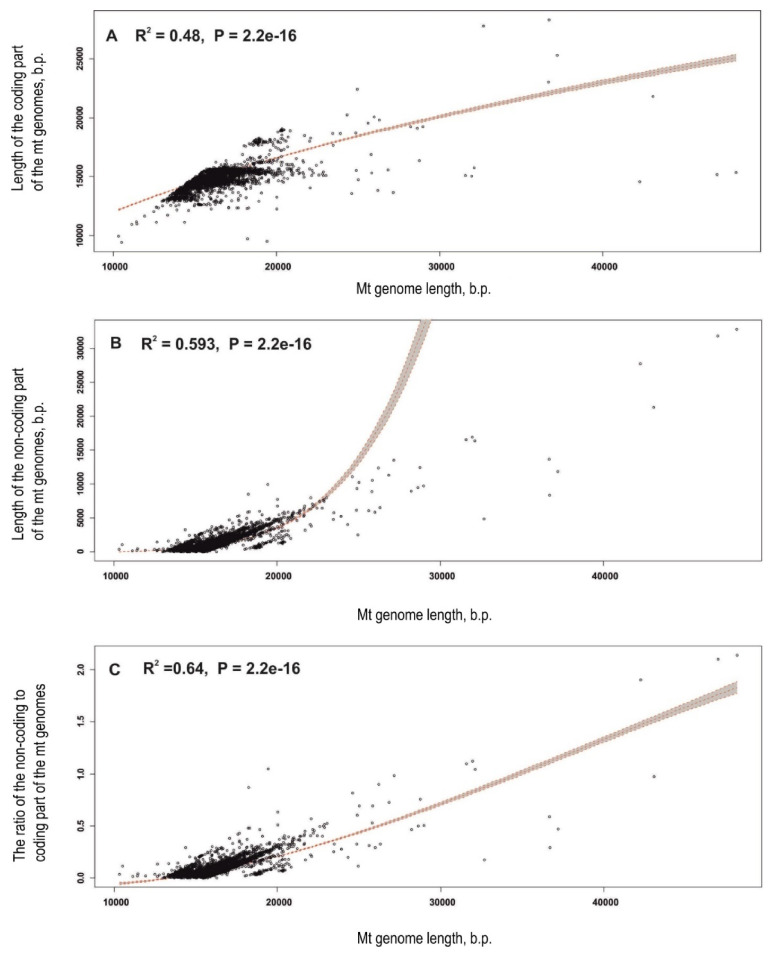
Visualization of the dependencies of mt genome lengths on the lengths of the entire mt genome assessed using regression analysis. (**A**) Dependence of coding lengths on the entire mt genome length. (**B**) Dependence of the non-coding lengths on the entire mt genome length. (**C**) Dependence of the ratio of non-coding to coding portions on the entire mt genome length. Regression curves are shown with a 95% confidence interval.

At the same time, we found a significant dependence of genome lengths on the phylum itself (*p*-value = 2 × 10 − 16, R2 from 0.963 to 0.999) using ANOVA (Table S11). A regression analysis revealed that the length of non-coding parts of mt genomes contributes more significantly (R2 = 0.592) to the entire mt genome length than the length of coding parts (R2 = 0.480) (Table S12). The longer the entire mt genome is, the larger the portion of its non-coding part will be (Figure 7, Table S12).

We also defined using ANOVA that the contribution to the variability of the entire mt genome length is more significant in mt genomes from the upward outliers category (F value = 2946) than in mt genomes from the downward outliers category (F value = 36.97) (Table S13). Figure S3 shows that the variability of the mt genome lengths of upward outliers is mainly determined by the variability of the lengths of the non-coding parts of the mt genomes (Table S13).

Thus, a statistical analysis and mt genome length distribution visualization showed that the length of the animal mt genomes, as well as the longest ones in different phyla (upward outliers), is mainly determined by the lengths of the non-coding parts, while the contribution of the coding part is much less.

### 3.5. Phylogenetic Analysis and Repeat Pattern Analysis of the Long Mt Genomes Sequences of Invertebrates

**Figure 8:**
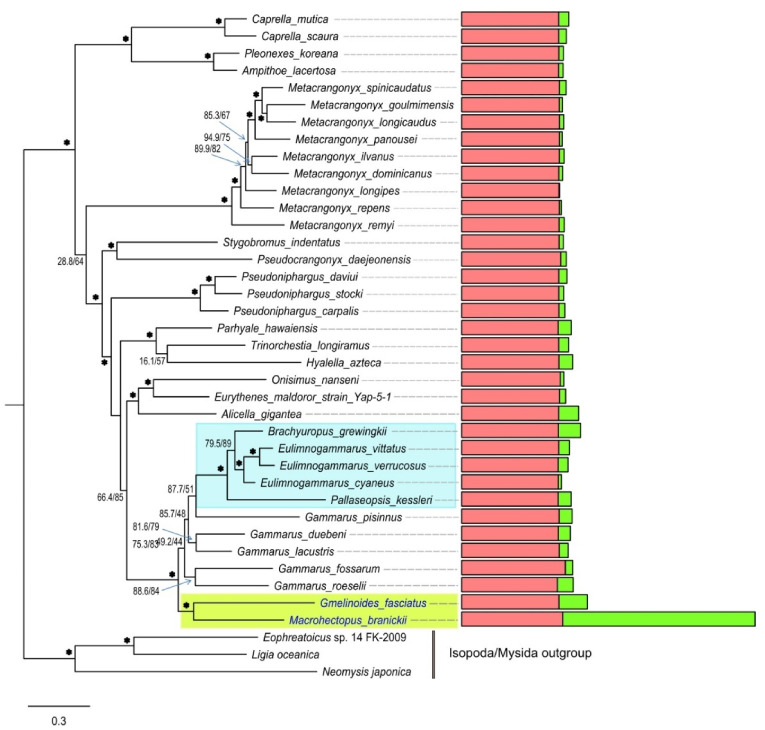
Maximum likelihood tree of Amphipoda species from the RefSeq database based on mt PCG amino acid sequences. Numbers at the branches indicate the percentage of ultrafast bootstrap and SH-aLRT, where values ≥ 95 are marked as asterisks. The horizontal histograms show the relative lengths of coding (red) and non-coding (green) regions of mt genomes in the corresponding species. Species whose non-coding region lengths fall into the category “outlier” are marked in blue. Yellow and blue rectangles show Baikalian amphipod species of the first and second lineages, respectively.

Mt genomes whose non-coding region lengths fall into the outliers category (the long mt genomes) were selected for phylogenetic analysis and repeat pattern examination. Phylogenetic trees were constructed using translated mt genome PCG sequences and included relatives from different taxa (Table S1) for the comparison of the mt genome lengths. We built nine phylogenetic trees encompassing the majority of outlier cases (Figure 8 and Figures S4–S11). Repeat patterns in long mt genomes are visualized in Figure S12.

We found a significant variability in the lengths of non-coding regions on the level of the large taxa and smaller taxa in particular, for instance, in Polychaeta and Amphipoda (phylum Arthropoda) species with long mt genomes cluster with species with smaller mt genomes. The topology of the Amphipoda tree (Figure 8) shows that a significant increase in the non-coding genome length occured in the lineage of *M. branickii*. Some groups of closely related species, such as representatives of superfamily Nephropoidea, subclass Copepoda, or species from gen. *Bombus* from order Hymenoptera (phylum Arthropoda), show a significant variability of mt non-coding region lengths (Figures S5 and S6). On the other hand, some groups, such as freshwater sponges (phylum Porifera), consistently maintain long non-coding regions in their mt genomes (Figure S11). Other examples of such groups include the Bivalvia mollusks of the Arcoidea superfamily (represented by *Cucullaea labiata*, *Scapharca broughtonii*, *Anadara sativa*, *Tegillarca granosa*) and nematodes of gen. *Meloigogyne*, clade of gen. *Trachelus*, and gen. *Cephus* in Hymenoptera (phylum Arthropoda) (Figures S5, S7, and S9).

Long mt genome sequences showed different patterns of repeats (Figure S12). The mt DNA sequences of some species from the phyla Annelida, Mollusca, and Nematodes possessed direct repeats with variable copy counts and lengths in the non-coding regions. The patterns with relatively long inverted repeats seen in *M. branickii* were rare and were found in only three Nematode species: *Hexamermis agrotis*, *Romanomermis culicivorax*, and *Romanomermis iyengari*. Very short tandem and inverted repeats constitute low-complexity regions in the non-coding regions of insects from order Hymenoptera, family Curculionidae, and species of gen. *Meloidogyne* from phylum Nematoda suborder Tylenchina. In all Porifera species with long non-coding regions, mt genome sequences have very short repeats covering the whole sequences almost evenly (Figure S12) [29,30]. The mt genomes of some species, such as *Longpotamon kenliense*, *Vespa affinis* (phylum Arthropoda), *Meloidogyne graminicola* (phylum Nematoda), and *Isodiametra pulchra* (phylum Xenacoelomorpha), did not show any repetitive sequences or had just a few very short repeats (Figure S12).

## 4. Discussion

### 4.1. The Unusual Architecture of Mt Genome of M. branickii and Its Potential Usefulness for Studies of Mt Genome Transcription and its Regulation

A newly sequenced mt genome of a pelagic amphipod *M. branickii* from Lake Baikal has an unusually large length of 42,256 bp, making this the largest length seen within Amphipods and one of the largest seen within all animals. Unusual gene orders and contents in comparison to other sequenced amphipod species were shown in a studied mt genome. In particular, there were duplications of four tRNA genes (*trnM*(cau)*, trnV*(uac)*, trnK*(uuu)*, trnD*(guc)), a *trnM*(cau)-derived pseudogene, and a partial copy of the *Cox2*. It is worth noting that all duplicated genes (tRNA genes and *Cox2*) are located on the opposite strand relative to their copy, which indicates that inversion events happened along with duplication. The full-length *Cox2* is located on the negative strand of the mt genome, which has never been observed in amphipods before and is not typical for the majority of mt genomes of Arthropods.

Additional tRNA genes and tRNA-like structures are sometimes found in mt genomes of different species, as well as in long mt genomes of some mollusks [20,46,116] and sponges [30]. Full-length and non-degraded copies of PCGs and ribosomal RNA genes are far less frequently identified in mt genomes than copies of tRNA genes; however, there are several examples of these cases. For instance, duplicated *Cox2* were found in the mt genomes of mollusks of the species *Ruditapes philippinarum* (in F-type) [63,65]; *Musculista senhousia* [16] and *Venustaconcha ellipsiformis* [117] (in M-type); and *Chaetoderma nitidulum* [118], *Anadara crebricostata*, *Scapharca inaequivalvis*, *Scapharca kagoshimensis*, and *Tegillarca* sp. [19]. Authors have suggested that there may be a different functional status of additional gene copies, such as an ongoing pseudogenization [65], neofunctionalization [116,119], or concerted evolution of the copies [17]. The partial duplication of the *Cox2* in the mt genome of *M. branickii* happened recently, as both 559 bp fragments were identical. At the same time, the adjacent *trnK*(uuu) and *trnD*(guc) had substitutions with their counterparts, as well as with other duplicated tRNA genes in the studied mt genome. The unusually high length of the non-coding region, multiple tRNA gene copies, and duplication of *Cox2* cause the mt genome of *M. branickii* to mostly resemble the long mt genomes of Bivalvia mollusks of the Arcidae family [19,20,60]. One of the possible reasons for the retention of gene copies and long non-coding regions in genomes is their potential functionality. For example, Li and colleagues proposed that the *Nad5*-derived non-coding fragment in the *Caenorhabditis briggsae* mt genome upregulates the transcription of the neighboring *Nad3* [120]. Transcribed copies of pseudogenes from non-coding regions of the nuclear and mt genomes were shown to participate in numerous processes of transcriptional and post-transcriptional gene regulation [121,122]. Moreover, proteomic studies confirmed the existence of small ORF-encoded peptides transcribed from non-coding regions or within PCGs or ribosomal RNA genes [123], affecting metabolism, development, DNA reparation, transcription, etc. [42,77]. tRNA genes, tRNA-like structures, and rRNA-derived fragments have also been shown to have a wide spectrum of functions beyond the mediation of translation [124,125]. However, most studies of such putative regulatory transcripts and peptides are devoted to nuclear-derived elements [77,78,121], and the regulatory potential of mt genome coding structures is yet to be assessed. Additionally, a detailed transcription pattern of the mt genome has been described in a handful of model organisms [123]. Thus, an unusual mt genome of *M. branickii* may be a useful model for studying the pattern of transcription and regulation of mt gene expression.

### 4.2. Features of the Long Mt Genomes in Invertebrates and Putative Mechanisms of Mt Genome Lengthening

About 65% of the length of the mt genome of *M. branickii* was annotated as non-coding intergenic regions. The two largest non-coding areas, interrupted by tRNA genes, are located between *Nad2* and *rrnL* and between *rrnS* and *Nad6*. A BLAST search revealed vestiges of ribosomal RNA genes inside these two regions, which nevertheless occupy only a minor part of the entire length of the non-coding area. Most of the two aforementioned non-coding regions consists of relatively long direct and inverted repeats. Such features of the non-coding region indicate that the mechanism for the extension of the mt genome of *M. branickii* involves multiple duplications and inversions of regions harboring ribosomal RNA genes, with the subsequent degradation of redundant gene copies. Phylogenetic analysis based on available complete mt genome sequences of amphipods shows that a significant mt genome length is a unique feature of *M. branickii*. It is also worth noting that the relatively long mt genomes of Baikalian amphipod species (>17 Kbp) do not display the repeat patterns observed in the sequence of *M. branickii*, i.e., there is no proliferation of ribosomal RNA genes. Thus, *M. branickii* has a unique pattern of mt genome extension among amphipods. It is worth mentioning that *M. branickii*, uniquely among Baikalian amphipods, has a pelagic lifestyle; however, it is not clear whether the length of the mt genome is specifically associated with this lifestyle. One of the possible approaches to studying this issue would be to analyze of the effective population size and other characteristics of populations of this species in Lake Baikal using mt genes as molecular markers. It would also be useful to assess the variability of mt genome lengths, as well as the integrity of duplicated genes in the mt genome of *M. branickii*, on the population level.

The statistical analysis showed that animal mt genome lengths and the presence of mt genomes with prominent expansions in sequence length (upward outliers in length distributions) depend on the phylum, but not on the number of sequences in the phylum, which refutes sample size bias as a significant factor in the assessment. Thus, a significant bias in the number of available mt genomes within different animal phyla may not be taken into consideration in studies of mt genome length patterns. It was also shown that the overall mt genome length mainly depends on the variability of their non-coding region. Although the latter conclusion was previously made by different authors based on the analysis of separate species and taxa [19,32,126], we confirmed that this is a common rule for animal mt genomes in general (Figure 7, Table S12).

Species with especially long mt genomes, including *M. branickii*, were included in the phylogenetic analysis. We detected different ranges for the length variability of the non-coding regions in mt genomes of different taxa. In some lineages (for example, superfamily Nephropoidea, subclass Copepoda, species from gen. *Bombus* from order Hymenoptera) (Figures S5 and S6), non-coding regions differ considerably, while other lineages maintain relatively short or unusually long non-coding regions for long evolutionary periods. Species from the latter group may be useful as models for studying the ecological reasons for and mechanisms of mt genome length extension and/or maintenance.

For instance, some species of Bivalvia mollusks from the family Arcidae have long mt genomes (Figure S7) [19]. Authors have shown that the time of divergence in a clade of gen. *Scapharca*, which includes species with the largest mt genome sizes (45.9–56.2 Kbp), was about 61 My and proposed that the low metabolic rate seen in these bivalves is associated with weakened purifying selection against long non-coding regions [19]. Another interesting group is freshwater sponges of the order Spongillida (Figure S11) [21,30,63,127], which split from marine sponges at about 18 My [128]. It is worth noting that within this group there are three endemic sponges from Lake Baikal (*Lubomirskia baikalensis*, *Rezinkovia echinata*, *Baikalospongia intermedia* morpha *profundalis*) [21,63]. Baikalian sponges are another example of Baikalian invertebrate species with long mt genomes. Among Baikalian invertebrates with sequenced mt genomes, there are also four endemic mollusk species from the family Baicaliidae that have mt genomes with lengths in the range of 15,127 to 15,224 bp. These mt genomes have a uniform gene order and very short non-coding regions [129]. Considering that Baicaliidae mollusks and Baikalian sponges are estimated to have comparable divergence times from their respective last common ancestors [128,129,130,131], further studies of the life histories of these species might hold clues for the reasons behind the variability of their mt genome lengths.

The majority of examined long mt genomes possess one or two long non-coding regions and a relatively compact cluster of PCGs and ribosomal genes. Such mt genome organization is frequently seen in mollusks, nematodes, insects, crustaceans species, etc. [46,132,133,134]. These non-coding regions often (but not always) contain repeat sequences (Figure S12). Relatively long direct repeats were identified in the mt genomes of mollusks, nematodes, and annelid species (Figure S12) [135,136,137,138]. Vestiges of different mt genes inside non-coding regions illuminate what parts of the genome were duplicated and degenerated, leading to mt genome length changes [46,139,140]. The non-coding regions of mt genomes of some species from different insects (order Hymenoptera, family Curculionidae) and Nematoda taxa (species of gen. *Meloidogyne*) possess segments with AT-rich short direct and inverted repeats (Figure S12), indicating their emergence from a duplicated control region [141,142]. The aforementioned repeat patterns suggest the contribution of the tandem duplication-random loss (TDRL) mechanism [143] in mt genome extension.

The large non-coding region of the mt genome of *M. branickii* possesses an approximately even ratio of relatively long direct and inverted repeats, which makes this pattern quite rare within the examined animal mt genomes. Indeed, the prevalence of direct repeats over inverted repeats in mt genomes was shown earlier in a study by Nardi and colleagues (2012) [71]. Patterns combining direct and inverted repeats were identified in only three nematode species: *R. iyengari*, *R. culicivorax* [18,144], and *H. agrotis* (Figure S12). The multiple sequence duplications and inversions found in the mt genomes of these species cannot be explained purely by the TDRL mechanism. An alternative explanation for such repeat patterns and the huge variation in the size of mt genomes could involve intramolecular or intermolecular recombination. Recombination in mt DNA was shown using bioinformatics and experimental approaches in plants, animals, and fungi [145,146,147,148,149], and, in particular, in the nematode *Meloidogyne javanica* [150]. Thus, recombination events along with duplications may have also contributed to the unusual mt genome architecture of *M. branickii*.

Another type of non-coding region repeat was detected in all analyzed long mt genomes of the phylum Porifera, which was, for the first time, noticed in the mt genome of *Suberites domuncula* [29]. Their short repeats are distributed almost evenly within mt genomes (Figure S12). Earlier studies of Porifera mt genomes carried out by Lavrov and colleagues [151] showed that the numerous non-coding regions seen in mt genomes maintain small repetitive palindromic sequences in different sponge species [21,30,63,151]. Authors have proposed that these elements do not have adaptive significance and evolve in mt genomes as selfish elements [21]. Similar types of intergenic repetitive elements (short inverted regions) were described in the animal mt genomes of the phylum Placozoa [32,61], as well as in mt genomes of algae and fungi [72,152].

Some lengthy mt genomes do not have repeats noted in species from different taxa, such as insects (*V. affinis*, Diadegma semiclausum, D. melanogaster, *Gonioctena intermedia*, etc.), annelids (Owenia fusiformis), Nematoda (M. graminicola), and crustaceans (Sinopotamon xiushuiense, L. kenliense) (Figure S12). We may assume that there were also duplication events in these genomes, but that the duplicated parts accumulated substantial substitutions or/and deletions and become indistinguishable as copies. Another but still less likely explanation for such sequence regions is their acquisition due to horizontal gene transfer, as was shown in Medusozoan Cnidarians [31], Octocorallia [34], and some Placozoan species [32]. The search for traces of horizontal gene transfer in the sequences of such mt genomes may well lead to new findings of unusual mt genes. It is also worth mentioning that the type of repeats seen, or their complete absence, is a lineage-specific feature, that is especially notable at the level of closely related species from monophyletic groups, such as gen. *Calameuta* (order Hymenoptera) or gen. *Metanephrops* (superfamily Nephropoidea) (Figures S4–S12). However, in the mt genomes of species from higher taxonomic units (superfamily, order, phylum), different types of repeats are frequently seen, that indicates duplication of different genome regions and different stages of their evolution.

Thus, the analysis of the huge number of invertebrate animal mt genomes points to at least two mechanisms that might be responsible for their extension, that were previously discussed in studies of smaller mt genome groups: (i) the duplication of different regions (both coding and non-coding) with the subsequent rapid degradation of redundant gene copies [143] and (ii) the proliferation of small palindromic intergenic sequences [21,63,151]. The large variation in the non-coding region lengths in comparison to the coding regions in the mt genomes implies reduced negative selection in the former group of sequences. The mt genome of the amphipod *M. branickii* may be a rare intermediate stage of mt genome evolution bearing signs of multiple duplications as sequence copies with different degrees of degeneration and an excess of the non-coding sequence that has not yet been deleted by selective pressure forces. Further studies of these sequence feature variations at the population level might provide us with clues as to their effect on organism fitness.

## 5. Conclusions

In this study, we report the complete mt genome of the pelagic amphipod species *M. branickii* from the ancient Lake Baikal. This mt genome has an unusually large length of 42,256 bp and a unique gene order within amphipods. In particular, duplications and inversions of four tRNA genes and *Cox2* were detected in the studied mt genome. The largest part of the mt genome consists of non-coding regions containing vestiges of ribosomal RNA genes. Phylogenetic analysis and the analysis of repeat patterns suggest that multiple duplications and inversions of regions containing ribosomal RNA genes occurred during the evolution of the *M. branickii* lineage and were followed by the degradation of redundant gene copies. The multiple inverted repeats found in the mt genome of *M. branickii* imply more complex mechanism of the sequence lengthening than mere duplication and loss. The mt genome of *M. branickii* is the largest found within amphipods so far and one of the largest within animals. We cofirmed that the length of animal mt genomes is mainly determined by the lengths of their non-coding regions. It was also revealed that mt genome length distributions depend on the phylum and are not affected by sampling bias. Thus, the emergence of exceptionally large mt genomes, such as that of *M. branickii*, is a rare and presumably lineage-specific phenomenon, as a more extensive sampling of other metazoan phyla did not reveal regular cases of large deviations in the lengths of mt genomes. Further studies on the details of *M. branickii* mt genome transcription, as well as the evolution of additional genes and long non-coding regions in populations of this species in Lake Baikal, will help us to define their degree of their evolution, maintenance and regulation.

## Data Availability

The data that support the findings of this study (sequencing reads and assemblies) are available from the corresponding authors upon reasonable request.

## Supplementary Materials

The following are available online at https://doi.org/10.6084/m9.figshare.16802182, Table S1: Data of the Maximum likelihood phylogenetic analyses of selected invertebrate taxa maintaining species whose non-coding mt genome region sizes were identified as outliers. File S1: Alignment of the Sanger sequence reads against the complete *M. branickii* mt genome. Table S2: The coverage statistics of the genomic and transcriptomic reads mapping on the mitochondrial genome of *M. branickii*. Table S3: The result of BlastN search in the mt genomes of Baikalian amphipods whose lengths exceed 17 Kbp. Table S4: The result of tRNA gene prediction in the mt genome of *M. branickii*. Figure S1: The predicted secondary structures of tRNA genes in the *M. branickii* mt genome. Figure S2: Dotplots of repeated sequences in mt genomes of Baikalian amphipods with lengths exceeding 17 Kbp. Table S5: Data on ORF findings in mt genomes of Baikalian amphipods with lengths exceeding 17 Kbp. Table S6: Data of the complete animal mt genomes retrieved from the RefSeq database submitted before 1 January 2020. Table S7: Statistical data of mt genome length characteristics in animal phyla sets. Table S8: RefSeq numbers of the long mt genomes whose non-coding region lengths fall into the category of upward outliers. Table S9: Dependence of the animal mt genomes length characteristics on the number of sequences in the phylum assessed using regression analysis. Table S10: Dependence of the portions of outliers in sequence sets of genome length characteristics in phyla on the number of sequences in the phyla assessed using regression analysis. Table S11: Dependence of animal mt genome length characteristics on belonging to a phylum assessed using ANOVA. Table S12: Dependence of mt genome length characteristics on the length of the entire mt genome assessed using regression analysis. Table S13: Dependencies of entire mt genome lengths on belonging to three gradations in ANOVA (mt genome lengths within general distributions, excluding values from outliers; mt genome lengths from downward outliers; mt genome lengths from upward outliers). Figure S3: Distributions of mt genome lengths from three sets: lengths of mt genomes within general distribution values (excluding values from outliers), lengths of mt genomes from downward outliers, and lengths of mt genomes from upward outliers shown for the lengths of the entire mt genome, lengths of coding mt genome parts, and lengths of non-coding mt genome parts. Figure S4: Maximum likelihood tree of Polychaeta species (phylum Annelida) from the RefSeq database based on mt PCG amino acid sequences. Figure S5: Maximum likelihood tree of Arthropods group 2 (Hymenoptera, Scarabaeiformia Staphyliniformia, Curculionidae, Nephropoidea) from the RefSeq database based on mt PCG amino acid sequences. Figure S6: Maximum likelihood tree of Arthropods group 3 (Copepoda, Potamoidea, Nephropoidea, Pentastomida) from the RefSeq database based on mt PCG amino acid sequences. Figure S7: Maximum likelihood tree of Pteriomorphia (phylum Mollusca) from the RefSeq database based on mt PCG amino acid sequences. Figure S8: Maximum likelihood tree of Dorylaimia (phylum Nematoda) from the RefSeq database based on mt PCG amino acid sequences. Figure S9: Maximum likelihood tree of order Strongylida and suborder Tylenchina (phylum Nematoda) from the RefSeq database based on mt PCG amino acid sequences. Figure S10: Maximum likelihood tree of phylum Xenacoelomorpha from the RefSeq database based on mt PCG amino acid sequences. Figure S11: Maximum likelihood tree of class Demospongiae (phylum Porifera) species from the RefSeq database based on mt PCG amino acid sequences. Figure S12: Dotplots of repeat sequences in long mt genomes of animals.

## References

[1] Smith D.R. (2016). The past, present and future of mitochondrial genomics: Have we sequenced enough mtDNAs?. Brief. Funct. Genom..

[2] Vasileiou P.V.S., Mourouzis I., Pantos C. (2017). Principal aspects regarding the maintenance of mammalian mitochondrial genome integrity. Int. J. Mol. Sci..

[3] Lavrov D.V., Pett W. (2016). Animal mitochondrial DNA as we do not know it: Mt-genome organization and evolution in nonbilaterian lineages. Genome Biol. Evol..

[4] Boore J.L. (1999). Animal mitochondrial genomes. Nucleic Acids Res..

[5] Breton S., Milani L., Ghiselli F., Guerra D., Stewart D.T., Passamonti M. (2014). A resourceful genome: Updating the functional repertoire and evolutionary role of animal mitochondrial DNAs. Trends Genet..

[6] Ladoukakis E.D., Zouros E. (2017). Evolution and inheritance of animal mitochondrial DNA: Rules and exceptions. J. Biol. Res. Thessaloniki.

[7] Shao Z., Graf S., Chaga O.Y., Lavrov D.V. (2006). Mitochondrial genome of the moon jelly *Aurelia aurita* (Cnidaria, Scyphozoa): A linear DNA molecule encoding a putative DNA-dependent DNA polymerase. Gene.

[8] Pan H.C., Fang H.Y., Li S.W., Liu J.H., Wang Y., Wang A.T. (2014). The complete mitochondrial genome of *Hydra vulgaris* (Hydroida: Hydridae). Mitochondrial DNA.

[9] Lavrov D.V., Pett W., Voigt O., Wörheide G., Forget L., Lang B.F., Kayal E. (2013). Mitochondrial DNA of *Clathrina clathrus* (Calcarea, Calcinea): Six linear chromosomes, fragmented rRNAs, tRNA editing, and a novel genetic code. Mol. Biol. Evol..

[10] Kim T., Kern E., Park C., Nadler S.A., Bae Y.J., Park J.K. (2018). The bipartite mitochondrial genome of *Ruizia karukerae* (Rhigonematomorpha, Nematoda). Sci. Rep..

[11] Song F., Li H., Liu G.H., Wang W., James P., Colwell D.D., Tran A., Gong S., Cai W., Shao R. (2019). Mitochondrial genome fragmentation unites the parasitic lice of Eutherian mammals. Syst. Biol..

[12] Suga K., Mark Welch D.B., Tanaka Y., Sakakura Y., Hagiwara A. (2008). Two circular chromosomes of unequal copy number make up the mitochondrial genome of the rotifer *Brachionus plicatilis*. Mol. Biol. Evol..

[13] Jühling F., Pütz J., Bernt M., Donath A., Middendorf M., Florentz C., Stadler P.F. (2012). Improved systematic tRNA gene annotation allows new insights into the evolution of mitochondrial tRNA structures and into the mechanisms of mitochondrial genome rearrangements. Nucleic Acids Res..

[14] Schirtzinger E.E., Tavares E.S., Gonzales L.A., Eberhard J.R., Miyaki C.Y., Sanchez J.J., Hernandez A., Müeller H., Graves G.R., Fleischer R.C. (2012). Multiple independent origins of mitochondrial control region duplications in the order Psittaciformes. Mol. Phylogenet. Evol..

[15] Williams S.T., Foster P.G., Hughes C., Harper E.M., Taylor J.D., Littlewood D.T.J., Dyal P., Hopkins K.P., Briscoe A.G. (2017). Curious bivalves: Systematic utility and unusual properties of anomalodesmatan mitochondrial genomes. Mol. Phylogenet. Evol..

[16] Passamonti M., Ricci A., Milani L., Ghiselli F. (2011). Mitochondrial genomes and Doubly Uniparental Inheritance: New insights from *Musculista senhousia* sex-linked mitochondrial DNAs (Bivalvia Mytilidae). BMC Genom..

[17] Yokobori S.I., Fukuda N., Nakamura M., Aoyama T., Oshima T. (2004). Long-term conservation of six duplicated structural genes in cephalopod mitochondrial genomes. Mol. Biol. Evol..

[18] Azevedo J.L., Hyman B.C. (1993). Molecular characterization of lengthy mitochondrial DNA duplications from the parasitic nematode *Romanomermis culicivorax*. Genetics.

[19] Kong L., Li Y., Kocot K.M., Yang Y., Qi L., Li Q., Halanych K.M. (2020). Mitogenomics reveals phylogenetic relationships of Arcoida (Mollusca, Bivalvia) and multiple independent expansions and contractions in mitochondrial genome size. Mol. Phylogenet. Evol..

[20] Liu Y.G., Kurokawa T., Sekino M., Tanabe T., Watanabe K. (2013). Complete mitochondrial DNA sequence of the ark shell *Scapharca broughtonii*: An ultra-large metazoan mitochondrial genome. Comp. Biochem. Physiol.-D Genom. Proteom..

[21] Lavrov D.V., Maikova O.O., Pett W., Belikov S.I. (2012). Small inverted repeats drive mitochondrial genome evolution in Lake Baikal sponges. Gene.

[22] Romanova E.V., Bukin Y.S., Mikhailov K.V., Logacheva M.D., Aleoshin V.V., Sherbakov D.Y. (2020). Hidden cases of tRNA gene duplication and remolding in mitochondrial genomes of amphipods. Mol. Phylogenet. Evol..

[23] Chong R.A., Mueller R.L. (2017). Polymorphic duplicate genes and persistent non-coding sequences reveal heterogeneous patterns of mitochondrial DNA loss in salamanders. BMC Genom..

[24] Kang H., Li B., Ma X., Xu Y. (2018). Evolutionary progression of mitochondrial gene rearrangements and phylogenetic relationships in Strigidae (Strigiformes). Gene.

[25] Wang X., Huang Y., Liu N., Yang J., Lei F. (2015). Seven complete mitochondrial genome sequences of bushtits (Passeriformes, Aegithalidae, Aegithalos): The evolution pattern in duplicated control regions. Mitochondrial DNA.

[26] Eberhard J.R., Wright T.F. (2016). Rearrangement and evolution of mitochondrial genomes in parrots. Mol. Phylogenet. Evol..

[27] Sayadi A., Immonen E., Tellgren-Roth C., Arnqvist G. (2017). The evolution of dark matter in the mitogenome of seed beetles. Genome Biol. Evol..

[28] Lavrov D.V., Forget L., Kelly M., Lang B.F. (2005). Mitochondrial genomes of two demosponges provide insights into an early stage of animal evolution. Mol. Biol. Evol..

[29] Lukić-Bilela L., Brandt D., Pojskić N., Wiens M., Gamulin V., Müller W.E.G. (2008). Mitochondrial genome of *Suberites domuncula*: Palindromes and inverted repeats are abundant in non-coding regions. Gene.

[30] Wang X., Lavrov D.V. (2008). Seventeen new complete mtDNA sequences reveal extensive mitochondrial genome evolution within the Demospongiae. PLoS ONE.

[31] Kayal E., Bentlage B., Collins A.G., Kayal M., Pirro S., Lavrov D.V. (2012). Evolution of linear mitochondrial genomes in medusozoan cnidarians. Genome Biol. Evol..

[32] Miyazawa H., Osigus H.J., Rolfes S., Kamm K., Schierwater B., Nakano H. (2021). Mitochondrial genome evolution of placozoans: Gene rearrangements and repeat expansions. Genome Biol. Evol..

[33] Pont-Kingdon G.A., Okada N.A., Macfarlane J.L., Beagley C.T., Wolstenholme D.R., Cavalier-Smith T., Clark-Walker G.D. (1995). A coral mitochondrial mutS gene. Nature.

[34] Bilewitch J.P., Degnan S.M. (2011). A unique horizontal gene transfer event has provided the octocoral mitochondrial genome with an active mismatch repair gene that has potential for an unusual self-contained function. BMC Evol. Biol..

[35] Shimpi G.G., Vargas S., Poliseno A., Wörheide G. (2017). Mitochondrial RNA processing in absence of tRNA punctuations in octocorals. BMC Mol. Biol..

[36] Szitenberg A., Rot C., Ilan M., Huchon D. (2010). Diversity of sponge mitochondrial introns revealed by *cox 1* sequences of Tetillidae. BMC Evol. Biol..

[37] Banguera-Hinestroza E., Ferrada E., Sawall Y., Flot J.F. (2019). Computational Characterization of the mtORF of Pocilloporid Corals: Insights into Protein Structure and Function in *Stylophora* Lineages from Contrasting Environments. Genes.

[38] Chi S.I., Urbarova I., Johansen S.D. (2018). Expression of homing endonuclease gene and insertion-like element in sea anemone mitochondrial genomes: Lesson learned from *Anemonia viridis*. Gene.

[39] Szafranski P. (2017). Evolutionarily recent, insertional fission of mitochondrial *cox2* into complementary genes in bilaterian Metazoa. BMC Genom..

[40] Breton S., Stewart D.T., Shepardson S., Trdan R.J., Bogan A.E., Chapman E.G., Ruminas A.J., Piontkivska H., Hoeh W.R. (2011). Novel protein genes in animal mtDNA: A new sex determination system in freshwater mussels (Bivalvia: Unionoida)?. Mol. Biol. Evol..

[41] Passamonti M., Calderone M., Delpero M., Plazzi F. (2020). Clues of in vivo nuclear gene regulation by mitochondrial short non-coding RNAs. Sci. Rep..

[42] Plaza S., Menschaert G., Payre F. (2017). In search of lost small peptides. Annu. Rev. Cell Dev. Biol..

[43] Pozzi A., Plazzi F., Milani L., Ghiselli F., Passamonti M. (2017). SmithRNAs: Could mitochondria “bend” nuclear regulation?. Mol. Biol. Evol..

[44] Aguado M.T., Grande C., Gerth M., Bleidorn C., Noreña C. (2016). Characterization of the complete mitochondrial genomes from Polycladida (Platyhelminthes) using next-generation sequencing. Gene.

[45] Zhang D., Li W.X., Zou H., Wu S.G., Li M., Jakovlić I., Zhang J., Chen R., Wang G.T. (2018). Mitochondrial genomes of two diplectanids (Platyhelminthes: Monogenea) expose paraphyly of the order Dactylogyridea and extensive tRNA gene rearrangements. Parasites Vectors.

[46] Wu X., Xu X., Yu Z., Wei Z., Xia J. (2010). Comparison of seven *Crassostrea* mitogenomes and phylogenetic analyses. Mol. Phylogenet. Evol..

[47] Palomares-Rius J.E., Cantalapiedra-Navarrete C., Archidona-Yuste A., Blok V.C., Castillo P. (2017). Mitochondrial genome diversity in dagger and needle nematodes (Nematoda: Longidoridae). Sci. Rep..

[48] Rosengarten R.D., Sperling E.A., Moreno M.A., Leys S.P., Dellaporta S.L. (2008). The mitochondrial genome of the hexactinellid sponge *Aphrocallistes vastus*: Evidence for programmed translational frameshifting. BMC Genom..

[49] Poliseno A., Feregrino C., Sartoretto S., Aurelle D., Wörheide G., McFadden C.S., Vargas S. (2017). Comparative mitogenomics, phylogeny and evolutionary history of *Leptogorgia* (Gorgoniidae). Mol. Phylogenet. Evol..

[50] Helfenbein K.G., Fourcade H.M., Vanjani R.G., Boore J.L. (2004). The mitochondrial genome of *Paraspadella gotoi* is highly reduced and reveals that chaetognaths are a sister group to protostomes. Proc. Natl. Acad. Sci. USA.

[51] Miyamoto H., Machida R.J., Nishida S. (2010). Complete mitochondrial genome sequences of the three pelagic chaetognaths *Sagitta nagae*, *Sagitta decipiens* and *Sagitta enflata*. Comp. Biochem. Physiol.-D Genom. Proteom..

[52] Arafat H., Alamaru A., Gissi C., Huchon D. (2018). Extensive mitochondrial gene rearrangements in Ctenophora: Insights from benthic Platyctenida. BMC Evol. Biol..

[53] Schultz D.T., Eizenga J.M., Corbett-Detig R.B., Francis W.R., Christianson L.M., Haddock S.H. (2020). Conserved novel ORFs in the mitochondrial genome of the ctenophore *Beroe forskalii*. PeerJ.

[54] Pett W., Ryan J.F., Pang K., Mullikin J.C., Martindale M.Q., Baxevanis A.D., Lavrov D.V. (2011). Extreme mitochondrial evolution in the ctenophore *Mnemiopsis leidyi*: Insight from mtDNA and the nuclear genome. Mitochondrial DNA.

[55] Barthélémy R.M., Seligmann H. (2016). Cryptic tRNAs in chaetognath mitochondrial genomes. Comput. Biol. Chem..

[56] Dermauw W., Vanholme B., Tirry L., Van Leeuwen T. (2010). Mitochondrial genome analysis of the predatory mite *Phytoseiulus persimilis* and a revisit of the *Metaseiulus occidentalis* mitochondrial genome. Genome.

[57] Egger B., Bachmann L., Fromm B. (2017). *Atp8* is in the ground pattern of flatworm mitochondrial genomes. BMC Genom..

[58] Gan H.M., Grandjean F., Jenkins T.L., Austin C.M. (2019). Absence of evidence is not evidence of absence: Nanopore sequencing and complete assembly of the European lobster (*Homarus gammarus*) mitogenome uncovers the missing *nad2* and a new major gene cluster duplication. BMC Genom..

[59] Wang H., Zhang S., Li Y., Liu B. (2010). Complete mtDNA of *Meretrix lusoria* (Bivalvia: Veneridae) reveals the presence of an atp8 gene, length variation and heteroplasmy in the control region. Comp. Biochem. Physiol.-D Genom. Proteom..

[60] Pu L., Liu H., Wang G., Li B., Xia G., Shen M., Yang M. (2019). Complete mitochondrial genome of the cockle *Anadara antiquata* (Linnaeus, 1758). Mitochondrial DNA Part B.

[61] Osigus H.J., Rolfes S., Herzog R., Kamm K., Schierwater B. (2019). *Polyplacotoma mediterranea* is a new ramified placozoan species. Curr. Biol..

[62] Signorovitch A.Y., Buss L.W., Dellaporta S.L. (2007). Comparative genomics of large mitochondria in placozoans. PLoS Genet..

[63] Lavrov D.V. (2010). Rapid proliferation of repetitive palindromic elements in mtDNA of the endemic Baikalian sponge *Lubomirskia baicalensis*. Mol. Biol. Evol..

[64] Ohta T. (1973). Slightly deleterious mutant substitutions in evolution. Nature.

[65] Ghiselli F., Milani L., Guerra D., Chang P.L., Breton S., Nuzhdin S.V., Passamonti M. (2013). Structure, transcription, and variability of metazoan mitochondrial genome: Perspectives from an unusual mitochondrial inheritance system. Genome Biol. Evol..

[66] Klucnika A., Ma H. (2019). A battle for transmission: The cooperative and selfish animal mitochondrial genomes. Open Biol. J..

[67] Lynch M., Koskella B., Schaack S. (2006). Mutation pressure and the evolution of organelle genomic architecture. Science.

[68] Boore J.L., Sankoff D., Nadeau J.H. (2000). The duplication/random loss model for gene rearrangement exemplified by mitochondrial genomes of deuterostome animals. Comparative Genomics.

[69] Levinson G., Gutman G.A. (1987). Slipped-strand mispairing: A major mechanism for DNA sequence evolution. Mol. Biol. Evol..

[70] Mjelle K.A., Karlsen B.O., Jorgensen T.E., Moum T., Johansen S.D. (2008). Halibut mitochondrial genomes contain extensive heteroplasmic tandem repeat arrays involved in DNA recombination. BMC Genom..

[71] Nardi F., Carapelli A., Frati F. (2012). Repeated regions in mitochondrial genomes: Distribution, origin and evolutionary significance. Mitochondrion.

[72] Smith D.R., Lee R.W. (2009). The mitochondrial and plastid genomes of *Volvox carteri*: Bloated molecules rich in repetitive DNA. BMC Genom..

[73] Selosse M.A., Albert B., Godelle B. (2001). Reducing the genome size of organelles favours gene transfer to the nucleus. Trends Ecol. Evol..

[74] Rand D.M. (1993). Endotherms, ectotherms, and mitochondrial genome-size variation. J. Mol. Evol..

[75] Rand D.M. (2011). Population genetics of the cytoplasm and the units of selection on mitochondrial DNA in *Drosophila melanogaster*. Genetica.

[76] Ma H., O’Farrell P.H. (2016). Selfish drive can trump function when animal mitochondrial genomes compete. Nat. Genet..

[77] Gusic M., Prokisch H. (2020). ncRNAs: New players in mitochondrial health and disease?. Front. Genet..

[78] Reynolds J.C., Bwiza C.P., Lee C. (2020). Mitonuclear genomics and aging. Hum. Genet..

[79] Ro S., Ma H.Y., Park C., Ortogero N., Song R., Hennig G.W., Zheng H., Lin Y.M., Moro L., Hsieh J.T. (2013). The mitochondrial genome encodes abundant small noncoding RNAs. Cell Res..

[80] Capt C., Passamonti M., Breton S. (2016). The human mitochondrial genome may code for more than 13 proteins. Mitochondrial DNA.

[81] Romanova E.V., Aleoshin V.V., Kamaltynov R.M., Mikhailov K.V., Logacheva M.D., Sirotinina E.A., Gornov A.Y., Anikin A.S., Sherbakov D.Y. (2016). Evolution of mitochondrial genomes in Baikalian amphipods. BMC Genom..

[82] Bazikalova A.Y., Vereshagin G.Y. (1945). Lake Baikal amphipods. Proceedings of the Baikal Limnological Station.

[83] Kamaltynov R.M., Timoshkin O.A., Proviz V.I., Sitnikova T.Y., Slugina Z.V., Melnik N.G. (2009). Amfipoda: Gammaroidea in Angara and Yenisei rivers. Index of Animal Species Inhabiting Lake Baikal and Its Catchment Area.

[84] Bekman M.Y., Afanasyeva E.L. (1977). Distribution and production of Macrohectopus in Lake Baikal. Proceedings of Limnological Institute.

[85] Naumova E.Y., Zaidykov I.Y., Makarov M.M. (2020). Recent quantitative values of *Macrohectopus branickii* (Dyb.) (amphipoda) from Lake Baikal. J. Gt. Lakes Res..

[86] Karnaukhov D.Y., Bedulina D.S., Kaus A., Prokosov S.O., Sartoris L., Timofeyev M.A., Takhteev V.V. (2016). Behaviour of Lake Baikal amphipods as a part of the night migratory complex in the Kluevka settlement region (South-Eastern Baikal). Crustaceana.

[87] Takhteev V.V., Karnaukhov D.Y., Govorukhina E.B., Misharin A.S. (2019). Diel vertical migrations of hydrobionts in the coastal area of Lake Baikal. Inland Water Biol..

[88] Rudstam L.G., Melnik N.G., Timoshkin O.A., Hansson S., Pushkin S.V., Nemov V. (1992). Diel dynamics of an aggregation of *Macrohectopus branickii* (Dyb.) (Amphipoda, Gammaridae) in the Barguzin Bay, Lake Baikal, Russia. J. Gt. Lakes Res..

[89] Doyle J.J., Dickson E.E. (1987). Preservation of plant samples for DNA restriction endonuclease analysis. Taxon.

[90] Bolger A.M., Lohse M., Usadel B. (2014). Trimmomatic: A flexible trimmer for Illumina sequence data. Bioinformatics.

[91] Bankevich A., Nurk S., Antipov D., Gurevich A.A., Dvorkin M., Kulikov A.S., Lesin V.M., Nikolenko S.I., Pham S., Prjibelski A.D. (2012). SPAdes: A new genome assembly algorithm and its applications to single-cell sequencing. J. Comput. Biol..

[92] Altschul S.F., Madden T.L., Schaffer A.A., Zhang J., Zhang Z., Miller W., Lipman D.J. (1997). Gapped BLAST and PSI-BLAST: A new generation of protein database search programs. Nucleic Acids Res..

[93] Naumenko S.A., Logacheva M.D., Popova N.V., Klepikova A.V., Penin A.A., Bazykin G.A., Etingova A.E., Mugue N.S., Kondrashov A.S., Yampolsky L.Y. (2017). Transcriptome-based phylogeny of endemic Lake Baikal amphipod species flock: Fast speciation accompanied by frequent episodes of positive selection. Mol. Ecol..

[94] Hall T.A. (1999). BioEdit: A user-friendly biological sequence alignment editor and analysis program for Windows 95/98/NT. Nucleic Acids Symp. Ser..

[95] Langmead B., Salzberg S.L. (2012). Fast gapped-read alignment with Bowtie 2. Nat. Methods.

[96] Milne I., Stephen G., Bayer M., Cock P.J.A., Pritchard L., Cardle L., Shaw P.D., Marshall D. (2013). Using Tablet for visual exploration of second-generation sequencing data. Brief. Bioinform..

[97] Krzywinski M., Schein J., Birol I., Connors J., Gascoyne R., Horsman D., Jones S.J., Marra M.A. (2009). Circos: An information aesthetic for comparative genomics. Genome Res..

[98] Quinlan A.R., Hall I.M. (2010). BEDTools: A flexible suite of utilities for comparing genomic features. Bioinformatics.

[99] Bernt M., Donath A., Juhling F., Externbrink F., Florentz C., Fritzsch G., Putz J., Middendorf M., Stadler P.F. (2013). MITOS: Improved de novo metazoan mitochondrial genome annotation. Mol. Phylogenet. Evol..

[100] Kerpedjiev P., Hammer S., Hofacker I.L. (2015). Forna (force-directed RNA): Simple and effective online RNA secondary structure diagrams. Bioinformatics.

[101] Greiner S., Lehwark P., Bock R. (2019). OrganellarGenomeDRAW (OGDRAW) version 1.3.1: Expanded toolkit for the graphical visualization of organellar genomes. Nucleic Acids Res..

[102] Kurtz S., Phillippy A., Delcher A.L., Smoot M., Shumway M., Antonescu C., Salzberg S.L. (2004). Versatile and open software for comparing large genomes. Genome Biol..

[103] Nguyen L.T., Schmidt H.A., von Haeseler A., Minh B.Q. (2014). IQ-TREE: A fast and effective stochastic algorithm for estimating maximum-likelihood phylogenies. Mol. Biol. Evol..

[104] Katoh K., Standley D.M. (2013). MAFFT multiple sequence alignment software version 7: Improvements in performance and usability. Mol. Biol. Evol..

[105] Abascal F., Zardoya R., Telford M.J. (2010). TranslatorX: Multiple alignment of nucleotide sequences guided by amino acid translations. Nucleic Acids Res..

[106] Kalyaanamoorthy S., Minh B.Q., Wong T.K., von Haeseler A., Jermiin L.S. (2017). ModelFinder: Fast model selection for accurate phylogenetic estimates. Nat. Methods.

[107] Guindon S., Dufayard J.F., Lefort V., Anisimova M., Hordijk W., Gascuel O. (2010). New algorithms and methods to estimate maximum-likelihood phylogenies: Assessing the performance of PhyML 3.0. Syst. Biol..

[108] Hoang D.T., Chernomor O., Von Haeseler A., Minh B.Q., Vinh L.S. (2017). UFBoot2: Improving the ultrafast bootstrap approximation. Mol. Boil. Evol..

[109] Rambaut A. FigTree-v1.4.3. http://tree.bio.ed.ac.uk/software/figtree.

[110] Kassambara A. Machine Learning Essentials: Practical Guide in R. STHDA. http://www.sthda.com/english/articles/38-regression-model-validation/158-regression-model-accuracy-metrics-r-square-aic-bic-cp-and-more/.

[111] Basso D., Pesarin F., Salmaso L., Solari A. (2009). Synchronized Permutation Tests in Two-way ANOVA. Permutation Tests for Stochastic Ordering and ANOVA.

[112] Xia X. (2017). DAMBE6: New tools for microbial genomics, phylogenetics, and molecular evolution. J. Hered..

[113] Bekman M.Y., Galaziy G.I. (1962). Ecology and production of *Micruropus possolskii* sow. and *Gmelinoides fasciatus* stebb. System and Ecology of Crustaceans of Lake Baikal.

[114] Timofeyev M.A., Shatilina J.M., Stom D.I. (2001). Attitude to temperature factor of some endemic amphipods from Lake Baikal and Holarctic *Gammarus lacustris* Sars, 1863: A comparative experimental study. Arthropoda Sel..

[115] Macdonald K.S., Yampolsky L., Duffy J.E. (2005). Molecular and morphological evolution of the amphipod radiation of Lake Baikal. Mol. Phylogenet. Evol..

[116] Smith D.R., Snyder M. (2007). Complete mitochondrial DNA sequence of the scallop *Placopecten magellanicus*: Evidence of transposition leading to an uncharacteristically large mitochondrial genome. J. Mol. Evol..

[117] Chapman E.G., Piontkivska H., Walker J.M., Stewart D.T., Curole J.P., Hoeh W.R. (2008). Extreme primary and secondary protein structure variability in the chimeric male-transmitted cytochrome c oxidase subunit II protein in freshwater mussels: Evidence for an elevated amino acid substitution rate in the face of domain-specific purifying selection. BMC Evol. Biol..

[118] Stöger I., Schrödl M. (2013). Mitogenomics does not resolve deep molluscan relationships (yet?). Mol. Phylogenet. Evol..

[119] Chakrabarti R., Walker J.M., Stewart D.T., Trdan R.J., Vijayaraghavan S., Curole J.P., Hoeh W.R. (2006). Presence of a unique male-specific extension of C-terminus to the cytochrome c oxidase subunit II protein coded by the male-transmitted mitochondrial genome of *Venustaconcha ellipsiformis* (Bivalvia: Unionoidea). FEBS Lett..

[120] Li R., Ren X., Bi Y., Ding Q., Ho V.W.S., Zhao Z. (2018). Comparative mitochondrial genomics reveals a possible role of a recent duplication of NADH dehydrogenase subunit 5 in gene regulation. DNA Res..

[121] Dietrich A., Wallet C., Iqbal R.K., Gualberto J.M., Lotfi F. (2015). Organellar non-coding RNAs: Emerging regulation mechanisms. Biochimie.

[122] Milligan M.J., Harvey E., Yu A., Morgan A.L., Smith D.L., Zhang E., Berengut J., Sivananthan J., Subramaniam R., Skoric A. (2016). Global intersection of long non-coding RNAs with processed and unprocessed pseudogenes in the human genome. Front. Genet..

[123] Barshad G., Marom S., Cohen T., Mishmar D. (2018). Mitochondrial DNA transcription and its regulation: An evolutionary perspective. Trends Genet..

[124] De Lay N.R., Garsin D.A. (2016). The unmasking of ‘junk’RNA reveals novel sRNAs: From processed RNA fragments to marooned riboswitches. Curr. Opin. Microbiol..

[125] Schimmel P. (2018). The emerging complexity of the tRNA world: Mammalian tRNAs beyond protein synthesis. Nat. Rev. Mol. Cell Biol..

[126] Lavrov D.V. (2007). Key transitions in animal evolution: A mitochondrial DNA perspective. Integr. Comp. Biol..

[127] Pleše B., Lukić-Bilela L., Bruvo-Mađarić B., Harcet M., Imešek M., Bilandžija H., Ćetković H. (2012). The mitochondrial genome of stygobitic sponge *Eunapius subterraneus*: mtDNA is highly conserved in freshwater sponges. Hydrobiologia.

[128] Schuster A., Vargas S., Knapp I.S., Pomponi S.A., Toonen R.J., Erpenbeck D., Wörheide G. (2018). Divergence times in demosponges (Porifera): First insights from new mitogenomes and the inclusion of fossils in a birth-death clock model. BMC Evol. Biol..

[129] Peretolchina T.E., Sitnikova T.Y., Sherbakov D.Y. (2020). The complete mitochondrial genomes of four Baikal molluscs from the endemic family Baicaliidae (Caenogastropoda: Truncatelloida). J. Molluscan Stud..

[130] Mats V.D., Shcherbakov D.Y., Efimova I.M. (2011). Late Cretaceous-Cenozoic history of the Lake Baikal depression and formation of its unique biodiversity. Stratigr. Geol. Correl..

[131] Zubakov D.Y., Sherbakov D.Y., Sitnikova T.Y. (1997). Analysis of phylogenetic relationships of endemic baikalian mollusks Baicaliidae family based on nucleotide sequences partial mitochondrial CO1 gene. Mol. Biol..

[132] Chen Y.J., Kim S., Wan X. (2021). Mitochondrial genomes of the *Dorcus velutinus* complex (Coleoptera: Lucanidae) with the large intergenic spacer showing unique short sequence repeats and their implications for systematics. J. Asia Pac. Entomol..

[133] Mohandas N., Pozio E., La Rosa G., Korhonen P.K., Young N.D., Koehler A.V., Hall R.S., Sternberg P.W., Boag P.R., Jex A.R. (2014). Mitochondrial genomes of *Trichinella* species and genotypes–A basis for diagnosis, and systematic and epidemiological explorations. Int. J. Parasitol..

[134] Wang Y.F., Xu S.X., Zhu C.C., Jia X.N., Zhou X.M., Zou J.X. (2020). The complete mitochondrial genome of the freshwater crab *Longpotamon kenliense* (Decapoda, Brachyura, Potamidae) with phylogenetic consideration. Crustaceana.

[135] Feng Y., Li Q., Yu H., Kong L. (2017). Complete mitochondrial genome sequence of *Cucullaea labiata* (Arcoida: Cucullaeidae) and phylogenetic implications. Genes Genom..

[136] Xu K., Kanno M., Yu H., Li Q., Kijima A. (2011). Complete mitochondrial DNA sequence and phylogenetic analysis of Zhikong scallop *Chlamys farreri* (Bivalvia: Pectinidae). Mol. Biol. Rep..

[137] Sun L., Zhuo K., Wang H., Song H., Chi W., Zhang L.H., Liao J. (2014). The complete mitochondrial genome of *Aphelenchoides besseyi* (Nematoda: Aphelenchoididae), the first sequenced representative of the subfamily Aphelenchoidinae. Nematology.

[138] Li Y., Kocot K.M., Schander C., Santos S.R., Thornhill D.J., Halanych K.M. (2015). Mitogenomics reveals phylogeny and repeated motifs in control regions of the deep-sea family Siboglinidae (Annelida). Mol. Phylogenet. Evol..

[139] Seixas V.C., de Moraes Russo C.A., Paiva P.C. (2017). Mitochondrial genome of the Christmas tree worm *Spirobranchus giganteus* (Annelida: Serpulidae) reveals a high substitution rate among annelids. Gene.

[140] Robertson H.E., Lapraz F., Egger B., Telford M.J., Schiffer P.H. (2017). The mitochondrial genomes of the acoelomorph worms *Paratomella rubra*, *Isodiametra pulchra* and *Archaphanostoma ylvae*. Sci. Rep..

[141] Korkmaz E.M., Budak M., Ördek M.N., Başıbüyük H.H. (2016). The complete mitogenomes of *Calameuta filiformis* (Eversmann, 1847) and *Calameuta idolon* (Rossi, 1794) (Hymenoptera: Cephidae): The remarkable features of the elongated A+ T rich region in Cephini. Gene.

[142] Humphreys-Pereira D.A., Elling A.A. (2014). Mitochondrial genomes of *Meloidogyne chitwoodi* and *M. incognita* (Nematoda: Tylenchina): Comparative analysis, gene order and phylogenetic relationships with other nematodes. Mol. Biochem. Parasitol..

[143] Boore J.L., Brown W.M. (1998). Big trees from little genomes: Mitochondrial gene order as a phylogenetic tool. Curr. Opin. Genet. Dev..

[144] Hyman B.C., Lewis S.C., Tang S., Wu Z. (2011). Rampant gene rearrangement and haplotype hypervariation among nematode mitochondrial genomes. Genetica.

[145] Dahal S., Dubey S., Raghavan S.C. (2018). Homologous recombination-mediated repair of DNA double-strand breaks operates in mammalian mitochondria. Cell. Mol. Life Sci..

[146] Davila J.I., Arrieta-Montiel M.P., Wamboldt Y., Cao J., Hagmann J., Shedge V., Xu Y.Z., Weigel D., Mackenzie S.A. (2011). Double-strand break repair processes drive evolution of the mitochondrial genome in *Arabidopsis*. BMC Biol..

[147] Tsaousis A.D., Martin D.P., Ladoukakis E.D., Posada D., Zouros E. (2005). Widespread recombination in published animal mtDNA sequences. Mol. Biol. Evol..

[148] Fritsch E.S., Chabbert C.D., Klaus B., Steinmetz L.M. (2014). A genome-wide map of mitochondrial DNA recombination in yeast. Genetics.

[149] Kraytsberg Y., Schwartz M., Brown T.A., Ebralidse K., Kunz W.S., Clayton D.A., Vissing J., Khrapko K. (2004). Recombination of human mitochondrial DNA. Science.

[150] Lunt D.H., Hyman B.C. (1997). Animal mitochondrial DNA recombination. Nature.

[151] Erpenbeck D., Voigt O., Wörheide G., Lavrov D.V. (2010). The mitochondrial genomes of sponges provide evidence for multiple invasions by Repetitive Hairpin-forming Elements (RHE). BMC Genom..

[152] Bullerwell C.E., Leigh J., Forget L., Lang B.F. (2003). A comparison of three fission yeast mitochondrial genomes. Nucleic Acids Res..

